# Supplementary material for: Influenza Vaccination in the Elderly in Three Cities in China: Current Status and Influencing Factors Under Different Funding Policies
Source: Vaccines (Basel). 2025 Nov 12;13(11):1158. doi: 10.3390/vaccines13111158 (PMC12656659; doi:10.3390/vaccines13111158)
Supplement: Supplementary file 1 [file vaccines-13-01158-s001.zip › File S1-Informed Consent Script (Verbal).pdf]

File S1: Informed consent statement (verbal)

Project Title: 2023–2024 Survey on Influenza Vaccination Among Seniors Aged 60 and Above

Introduction:

Dear Sir/Madam,

Hello! Influenza is a respiratory infectious disease caused by the influenza virus that poses a serious threat to human health. Annual influenza vaccination is an effective means of prevention, and it can significantly reduce the risk of contracting influenza and the development of severe complications. The elderly are one of the high-risk groups for severe influenza. China's "Influenza Vaccine Prevention Technical Guidelines" list adults aged 60 and above as a priority group for vaccination. Although the government has implemented many measures to promote vaccination among high-risk groups, the influenza vaccination rate among the elderly in China has not seen a significant increase. Therefore, the Chinese Center for Disease Control and Prevention plans to conduct a survey on influenza vaccination among adults aged 60 and above to provide a scientific basis for proposing and implementing targeted measures to promote vaccination. This survey will not disclose personal information, and only aggregated data will be used for better vaccination policy formulation. You may skip any questions and unconditionally stop the survey at any time without any consequences.

If you have any questions or suggestions regarding the above content, you may contact the project team at: [xxx-xxxxxx].

Thank you for your cooperation!

Verbal Consent Script (To be read by investigator):

"I have read and fully understood the above information regarding this survey. I agree to voluntarily participate in this survey."

(Investigator records participant's verbal agreement and the date.)
