# Supplementary material for: Influenza Vaccination in the Elderly in Three Cities in China: Current Status and Influencing Factors Under Different Funding Policies
Source: Vaccines (Basel). 2025 Nov 12;13(11):1158. doi: 10.3390/vaccines13111158 (PMC12656659; doi:10.3390/vaccines13111158)
Supplement: Supplementary file 1 [file vaccines-13-01158-s001.zip › File S2- Survey Questionnaire.pdf]

File S2: The survey questionnaire (English translation)

Project Title: 2023–2024 Survey on Influenza Vaccination Among Seniors Aged 60 and Above

Part 1: Basic Information

1.Gender:

① Male

② Female

2. Age: ____ years

3. Marital Status:

① Unmarried

② Married/Partnered

③ Divorced

④ Widowed

4. Current living situation:

① Living alone

② Living only with spouse/partner

③ Living only with children

④ Living with spouse/partner and children

⑤ Residing in a nursing home/care facility

5. Education level:

① No formal education

② Primary school

③ Junior high school

④ Technical secondary/High school

⑤ College

⑥ University and above

6. Health insurance type:

① Urban Employee Basic Medical Insurance

② Urban and Rural Resident Basic Medical Insurance

③ Government Medical Scheme

④ No medical insurance

⑤ Other (Please specify) ______

7. Average monthly family income:

① <¥1000/month

② ¥1000–2999/month

③ ¥3000–4999/month

④ ¥5000–9999/month

⑤ ≥¥10,000/month

8. Do you have any chronic disease(s) (excluding simple hypertension)?

① Yes, ______ (Disease name)

② No

9. Do any of your co-resident family members have chronic disease(s) (excluding simple hypertension)?

① Yes

(Relationship) ______ (Disease name)

(Relationship) ______ (Disease name)

(Relationship) ______ (Disease name)

② No

Part 2: Vaccination Status and Policy Awareness

10. Did you receive the influenza vaccine for the 2023–2024 season (Aug. 2023–Apr. 2024)?

① Yes

② No

③ Not sure

11. Are you willing to receive the influenza vaccine for the next season (2024–2025, Aug. 2024–Apr. 2025)?

① Yes

② No

12. Have you ever received the pneumococcal vaccine?

① Yes

② No

③ Not sure

13. Have you received the COVID-19 vaccine?

① Yes, ____ dose(s)

② No

③ Not sure

14. a. Policy Awareness (Chengdu only): Starting from 2023, individuals aged ≥60 in Chengdu cannot use medical insurance to pay for influenza vaccination.

① Yes

② No

③ Not sure

b. Policy Awareness (Jiaxing only): Starting from 2020, permanent residents aged ≥60 or ≥70 in Jiaxing can receive influenza vaccination for free.

① Yes

② No

③ Not sure

15. Policy Awareness (Chengdu only): Starting from 2015, individuals aged ≥60 with Chengdu household registration can receive pneumococcal vaccination for free.

① Yes

② No

③ Not sure

Part 3: Health Belief Model Scale for Influenza Vaccination Influencing Factors

Basic Knowledge

16.Influenza is a respiratory infectious disease that can spread through coughing, sneezing, or talking.

① Strongly Agree ② Agree ③ Neutral ④ Disagree ⑤ Strongly Disagree

17. Influenza is just a common cold.

① Strongly Agree ② Agree ③ Neutral ④ Disagree ⑤ Strongly Disagree

18. The influenza vaccine needs to be administered annually.

① Strongly Agree ② Agree ③ Neutral ④ Disagree ⑤ Strongly Disagree

Perceived Susceptibility

19.Your likelihood of getting influenza increases during winter/spring.

① Strongly Agree ② Agree ③ Neutral ④ Disagree ⑤ Strongly Disagree

20. Compared to younger people, you are more susceptible to influenza.

① Strongly Agree ② Agree ③ Neutral ④ Disagree ⑤ Strongly Disagree

21. Based on your current health status, you are susceptible to influenza.

① Strongly Agree ② Agree ③ Neutral ④ Disagree ⑤ Strongly Disagree

22. You might have already had influenza in the past few months.

① Strongly Agree ② Agree ③ Neutral ④ Disagree ⑤ Strongly Disagree

23. You worry about getting influenza during the next winter/spring season.

① Strongly Agree ② Agree ③ Neutral ④ Disagree ⑤ Strongly Disagree

Perceived Severity

24.Influenza is a very serious illness.

① Strongly Agree ② Agree ③ Neutral ④ Disagree ⑤ Strongly Disagree

25. If you got influenza, your body would feel very unwell.

① Strongly Agree ② Agree ③ Neutral ④ Disagree ⑤ Strongly Disagree

26. If you got influenza, your daily activities would be affected.

① Strongly Agree ② Agree ③ Neutral ④ Disagree ⑤ Strongly Disagree

27. The thought of potential influenza symptoms makes you feel anxious, fearful, or tense.

① Strongly Agree ② Agree ③ Neutral ④ Disagree ⑤ Strongly Disagree

28.Influenza can lead to serious complications.

① Strongly Agree ② Agree ③ Neutral ④ Disagree ⑤ Strongly Disagree

Perceived Benefits

29.Influenza vaccination can prevent influenza.

① Strongly Agree ② Agree ③ Neutral ④ Disagree ⑤ Strongly Disagree

30. Influenza vaccination can reduce your risk of getting influenza or developing influenza-related complications.

① Strongly Agree ② Agree ③ Neutral ④ Disagree ⑤ Strongly Disagree

31. Getting vaccinated is a good idea because it reduces your worry about influenza infection.

① Strongly Agree ② Agree ③ Neutral ④ Disagree ⑤ Strongly Disagree

32. If you get vaccinated, it can reduce the number of your hospital visits (outpatient, emergency, and hospitalization) and associated medical costs.

① Strongly Agree ② Agree ③ Neutral ④ Disagree ⑤ Strongly Disagree

33. Even if you still get infected after vaccination, your symptoms will be much milder.

① Strongly Agree ② Agree ③ Neutral ④ Disagree ⑤ Strongly Disagree

Perceived Barriers

34.Getting the influenza vaccine is very painful (during the injection).

① Strongly Agree ② Agree ③ Neutral ④ Disagree ⑤ Strongly Disagree

35. The influenza vaccine can cause influenza.

① Strongly Agree ② Agree ③ Neutral ④ Disagree ⑤ Strongly Disagree

36. The side effects of the influenza vaccine would affect your daily activities.

① Strongly Agree ② Agree ③ Neutral ④ Disagree ⑤ Strongly Disagree

37. The influenza vaccine does not prevent influenza.

① Strongly Agree ② Agree ③ Neutral ④ Disagree ⑤ Strongly Disagree

38. The side effects of the influenza vaccine are worse than influenza itself.

① Strongly Agree ② Agree ③ Neutral ④ Disagree ⑤ Strongly Disagree

39. You are afraid of injections.

① Strongly Agree ② Agree ③ Neutral ④ Disagree ⑤ Strongly Disagree

40. The cost of the influenza vaccine is too high.

① Strongly Agree ② Agree ③ Neutral ④ Disagree ⑤ Strongly Disagree

41. It is difficult to schedule an appointment for influenza vaccination.

① Strongly Agree ② Agree ③ Neutral ④ Disagree ⑤ Strongly Disagree

42. Do you know where to get the influenza vaccine?

① Yes

② Not sure

③ No

Estimated distance from your home to the nearest vaccination point: ______ km

43. Transportation to the influenza vaccination site is inconvenient.

① Strongly Agree ② Agree ③ Neutral ④ Disagree ⑤ Strongly Disagree

44. You don't have time to go to the vaccination site.

① Strongly Agree ② Agree ③ Neutral ④ Disagree ⑤ Strongly Disagree

Cues to Action

45.How easy is it for you to obtain information about influenza and the influenza vaccine?

① Very Easy ② Easy ③ Neutral ④ Difficult ⑤ Very Difficult

Which platforms have you used to learn about influenza/vaccine information? (Multiple choice):

① Community bulletin boards/posters

② Community doctor face-to-face

③ Mobile phone

④ Radio/TV

⑤ Family/friends

⑥ Other ______ (Please specify)

46. If your family wanted you to get vaccinated, you would choose to do so.

① Strongly Agree ② Agree ③ Neutral ④ Disagree ⑤ Strongly Disagree

47. If friends, colleagues, or neighbors wanted you to get vaccinated, you would choose to do so.

① Strongly Agree ② Agree ③ Neutral ④ Disagree ⑤ Strongly Disagree

48. If healthcare workers recommended vaccination, you would choose to do so.

① Strongly Agree ② Agree ③ Neutral ④ Disagree ⑤ Strongly Disagree

(If "Neutral," "Agree," or "Strongly Agree," please rank the following by acceptance level, from highest to lowest):

______ > ______ > ______ > ______ > ______

① Community health center staff

② Hospital staff (secondary level and above)

③ CDC staff

④ Renowned scholars/experts in the healthcare field

⑤ Other ______ (Please specify)

49. If government officials, celebrities, or other public figures recommended vaccination, you would choose to do so.

① Strongly Agree ② Agree ③ Neutral ④ Disagree ⑤ Strongly Disagree

If reminded via text message, community notice, poster, or WeChat group, you would be more willing to get vaccinated.

① Strongly Agree ② Agree ③ Neutral ④ Disagree ⑤ Strongly Disagree

50. How do you prefer to receive information about influenza/vaccines/policies? (Select 4, rank by preference from highest to lowest):

______ > ______ > ______ > ______

① Posters/flyers/banners

② Public service ads/short videos/songs/raps/rhymes

③ Official websites/articles from professional institutions

④ Face-to-face communication from community/CDC/hospital/school staff

⑤ Other ______ (Please specify)

End of Survey. Thank you for your participation!
